# Bone marrow-derived neural crest precursors improve nerve defect repair partially through secreted trophic factors

**DOI:** 10.1186/s13287-019-1517-1

**Published:** 2019-12-18

**Authors:** Haiyan Shi, Xiaoli Li, Junling Yang, Yahong Zhao, Chengbin Xue, Yaxian Wang, Qianru He, Mi Shen, Qi Zhang, Yumin Yang, Fei Ding

**Affiliations:** 1 Department of Pathophysiology, School of Medicine, Nantong University, 19 Qixiu Road, Nantong, 226001 China; 2 Key Laboratory of Neuroregeneration of Jiangsu and Ministry of Education and Co-innovation Center of Neuroregeneration, 19 Qixiu Road, Nantong, 226001 China; 3 Department of Pathology, Affiliated Hospital of Nantong University, 20 Xisi Road, Nantong, 226001 China; 4 Research Center of Clinical Medicine, Affiliated Hospital of Nantong University, 20 Xisi Road, Nantong, 226001 China; 5 Jiangsu Clinical Medicine Center of Tissue Engineering and Nerve Injury Repair, Affiliated Hospital of Nantong University, 20 Xisi Road, Nantong, 226001 China

**Keywords:** Neural crest, Precursor, Schwann cell, Nerve regeneration, Trophic factor, Bone marrow, Tissue engineering nerve graft

## Abstract

**Background:**

Emerging evidence suggests that neural crest-derived cells (NCCs) present important functions in peripheral nerve regeneration to correct the insufficiency of autogenous Schwann cells. Postmigratory NCCs have been successfully isolated from adult rat bone marrow in our previous work. In this study, we aim to provide neural crest-derived Schwann cell precursors (SCPs) for repair of nerve defects in adult rats, and partially reveal the mechanisms involved in neuroregeneration of cell therapy.

**Methods:**

A clonal cell line of neural crest precursors of rat bone marrow origin (rBM-NCPs) with SCP identity was expanded in adherent monolayer culture to ensure the stable cell viability of NCPs and potentiate the repair of nerve defects after rBM-NCPs implantation based on tissue engineering nerve grafts (TENG). Here the behavioral, morphological, and electrophysiological detection was performed to evaluate the therapy efficacy. We further investigated the treatment with NCP-conditioned medium (NCP-CM) to sensory neurons after exposure to oxygen-glucose-deprivation (OGD) and partially compared the expression of trophic factor genes in rBM-NCPs with that in mesenchymal stem cells of bone marrow origin (rBM-MSCs).

**Results:**

It was showed that the constructed TENG with rBM-NCPs loaded into silk fibroin fiber scaffolds/chitosan conduits repaired 10-mm long sciatic nerve defects more efficiently than conduits alone. The axonal regrowth, remyelination promoted the reinnervation of the denervated hind limb muscle and skin and thereby alleviated muscle atrophy and facilitated the rehabilitation of motor and sensory function. Moreover, it was demonstrated that treatment with NCP-CM could restore the cultured primary sensory neurons after OGD through trophic factors including epidermal growth factor (EGF), platelet-derived growth factor alpha (PDGFα), ciliary neurotrophic factor (CNTF), and vascular endothelial growth factor alpha (VEGFα).

**Conclusions:**

In summary, our findings indicated that monolayer-cultured rBM-NCPs cell-based therapy might effectively repair peripheral nerve defects partially through secreted trophic factors, which represented the secretome of rBM-NCPs differing from that of rBM-MSCs.

## Background

Schwann cell precursors (SCPs) are usually derived from neural crest cells (NCCs), emerged in contact with neuronal axons during peripheral nerve development [1]. Although neural crest is the transient structure in vertebrate embryos, cells with neural crest features can be found from non-neural crest tissues of adulthood and may differ in development anatomic location origin [2]. Post-migratory NCCs may resident in bone marrow, dorsal root ganglia (DRG), whisker pad, etc. [3].

SCPs present many important functions for peripheral nerve development and regeneration, including neuronal survival, axonal growth, myelination, nerve fasciculation, and target innervation. They may differentiate into various neural cells (e.g., glia, endoneurial fibroblasts, visceral neurons) and non-neural-like cells (e.g., mesenchymal stromal cells) [4]. Besides, SCPs are implicated in the regeneration of multiple tissue types such as skin and digit tip [5–7]. In the process of peripheral nerve regeneration, Schwann cells may orchestrate the regenerative homeostasis response [8]. The stimulated Schwann cells can respond to injury signals and dedifferentiate into repair type Schwann cells via reprogramming, present in a proliferating “macrophage-like” and “progenitor-like” state. The repair type Schwann cells enable to control the infiltration of inflammatory cells across the blood-nerve barrier, clear myelin, and axonal debris; regulate the extracellular matrix balance; and produce cytokines to guide axonal regrowth across the nerve gap.

However, it is hard to solve the regeneration problem of long-distance nerve defects or thick nerve injury resulted from the insufficiency of Schwann cell number. In addition, the damaged nerves clear debris more slowly in aged animals than in young animals due to diminished Schwann cell repair response [9, 10]. Therefore several stem cells and their derivatives are notable in the replacement of Schwann cells seeded in conduits to create tissue-engineered nerve grafts (TENG), which is considered as a promising alternative to autograft nerve transplant [11]. Mesenchymal stem cells (MSCs) have been supplemented into silk fibroin fiber scaffolds/chitosan conduits to construct TENG for bridging nerve gap in rats, dogs, and rhesus monkeys [12–15]. More recently, SCPs produced from human-induced pluripotent stem cells (iPSCs) were reported as a potential therapeutic target for myelin repair in mice [16]. Both SCPs and MSCs may originate from neural crest precursors (NCPs) [17].

Accordingly, we hypothesized that NCPs of rat bone marrow origin (rBM-NCPs) could serve as the source of seeding cells for the construction of TENG to repair sciatic nerve defects in adult rats. We previously focused on the isolation of rBM-NCPs subpopulation based on the evidence of NCCs resident in bone marrow [3], and bone marrow stromal cell-derived spheres cultured in vitro had been identified as neural crest-derived SCPs in terms of molecular phenotype. And SCP cellular behavior is characterized by differentiation potency, self-renewal capacity, and myelination ability, etc. [18]. The established clonal rBM-NCPs cell line N1 was named as rBMNCPN1 and will have been stored in the Preservation Center of Biological Preparations of Wuhan University in China for 30 years from 2016 (NO. 2016047).

In this study, we introduced rBM-NCPs into conduits to create TENG for bridging 10-mm-long distance gap of rat sciatic nerve. To evaluate the effectiveness of TENG transplantation therapy, the recovery of sensory and motor function after nerve repair was assessed within 12 weeks and was further detected by electrophysiology test and fluorescent retrograde axonal labeling technique. Simultaneously, the remyelination of regenerated nerve fibers and the morphology of hind limb muscles were observed by transmission electrical microscope (TEM). Additionally, the damage of adult rat DRG neurons in ischemic condition after sciatic nerve injury in vivo was simulated by oxygen-glucose-deprivation (OGD) of cultured primary neurons in vitro; then, injured neurons were treated with NCP-conditioned medium (NCP-CM). The expression of multiple trophic factors were analyzed in rBM-NCPs. Collectively, we investigated the repair potential and action mechanism of rBM-NCPs using nerve defect model in vivo and neuron injury model in vitro.

## Methods

### Preparation of rBM-NCPs and rBM-MSCs

We have established rBM-NCPs cell line and rBM-MSCs cell line in previous work [18, 19]. Cryopreserved rBM-NCPs spheres at passage 31 (P31) to P33 were resuscitated for proliferating in DMEM/F12 medium (v/v, 1:1, Gibco, Gaithersburg, MD) supplemented with 1% N2 (Gibco, Grand Island, NY), 2% B27 (Gibco), 20 ng/mL epidermal growth factor (EGF), and 20 ng/mL fibroblast growth factor-2 (FGF2) (both from R&D, Minneapolis, MN); big floating rBM-NCPs spheres were dispersed and formed single-cell suspension, then adherently sub-cultured on PLL (Sigma, St. Louis, MO)-coated culture ware at a density of 40,000 cells/cm^2^. Cryopreserved rBM-MSCs clone16 at P101-P103 were recovered for expansion and passaged in DMEM/F12 (1:1) medium supplemented with 10% fetal bovine serum (FBS, Gibco).

### Differentiation of rBM-NCPs into Schwann cells

The rBM-NCPs were dissociated into single cells, and cells were reinoculated on poly-l-lysine (PLL)/laminin (Invitrogen, Carlsbad, CA)-coated coverslips for incubation in differentiation medium. The medium, consisting of DMEM/F12 (1:1) supplemented with 5% FBS, 5 μM all-trans-retinoic acid (tRA, Sigma), 1% N2, 10 ng/mL FGF2, 10 ng/mL platelet-derived growth factor-AA (PDGF-AA, R&D), and 200 ng/mL β-heregulin 1 (HRG-1β, R&D), was changed every 3 days.

### Immunofluorescent staining

Monolayer-cultured rBM-NCPs were fixed with 4% (wt/vol) paraformaldehyde, blocked, and then incubated with primary antibodies, including rabbit-anti-CD133 (1:200, Abcam), rabbit-anti-p75 (1:1000, Cell Signaling), rabbit-anti-nestin (1:50, Sigma), mouse-anti-vimentin (1:25, Sigma), mouse-anti-CD29 (1:100, Sigma), and rabbit-anti-Ki67 (1:200, Sigma), overnight at 4 °C, followed by reaction with FITC-anti-rabbit-IgM (Sigma), Cy3-anti-rabbit-IgM (Santa Cruz), TRITC-anti-mouse-IgM (Santa Cruz) or FITC-anti-mouse-IgM (Sigma), and Hoechst 33342 (Sigma) counterstaining. The cell samples were observed under a confocal laser scanning microscope (TCS SP2, Leica Microsystems, Germany).

Induced Schwann cells were subjected to immunofluorescent staining with mouse anti-S100β (1:250, Sigma), rabbit-anti-glial fibrillary acidic protein (anti-GFAP, 1:200, DakoCytomation), and rabbit-anti-p75 (1:1000, Cell Signaling Technology) respectively, followed by the same reaction with the second antibody and Hoechst 33342 counterstaining.

### Label of rBM-NCPs for tracking in vivo after TENG transplantation

Adherently cultured rBM-NCPs on PLL-coated 35-mm dish at a density of 1.6 × 10^5^ cells were incubated in a 37 °C, 5% CO_2_ incubator overnight, followed by labeling with Qdot-tracker 565 (Invitrogen) for 60 min, then washed twice with complete growth medium before observation. Labeled cells were collected as supporting cells via Accutase (Sigma) digestion for construction of TENG. After TENG implantation into rat sciatic nerve defects for 7 days, the TENG samples were harvested, post-fixed, and cut into longitudinal sections on a cryostat, then labeled rBM-NCPs in conduits were observed.

### Preparation of silk fibroin fiber scaffolds/chitosan conduits

A chitosan conduit (inner diameter, 2.0 mm) was prepared as described previously [20] (please refer to Chinese patent ZL 0110820.9 for technical details). Silk fibroin fibers (diameter, 8 μm), prepared as described previously from *Bombyx mori* silk through a degumming process of boiling in aqueous sodium carbonate solution [21], were sheared into 15 mm long. To fabricate the silk fibroin fiber scaffolds/chitosan conduits, 5 silk fibroin fibers were inserted into the lumen of 10-mm long chitosan conduits.

### Construction of TENG and bridging of sciatic nerve defects

All experimental procedures involving animals were performed as the institutional animal care guidelines and ethically approved by the Administration Committee of Experimental Animals, Jiangsu Province, China.

The surgical procedure was conducted as previously described [22]. Adult male Wistar rats (8 weeks old, male, weighted 200–220 g, *n* = 12 per treatment group) were randomized into 3 grafted groups and a non-grafted group. The left sciatic nerve was exposed after a skin incision and muscles splitting after anesthesia with 10% chloral hydrate (0.3 mL/100 g), then a segment of sciatic nerve was transected and removed, leaving a 10-mm long gap following retraction of the nerve ends. In the conduit group, the grafts were silk fibroin fiber scaffolds/chitosan conduits filled with 0.1 M phosphate buffer (PBS, pH 7.4); in the TENG group, the conduit lumen was filled with 40–50 μl rBM-NCPs single-cell suspension at the density of 1 × 10^7^ cells/mL; in the autograft group, the transected nerve segment was implanted into the sciatic nerve gap in reversal direction; and in the non-grafted group, the sciatic nerve gap was left un-bridged. Postoperative rats were housed for 12 weeks and fed routinely. And rats were euthanized by overdose anesthesia with 10% chloral hydrate (0.6 mL/100 g) after harvesting implants.

### SFI detection and withdrawal reflex detection

Walking track analysis was performed to 4 groups 4, 6, 8, 10, and 12 weeks after surgery as previously described [23], and the sciatic function index (SFI) value was calculated by the formula of Bain group [24].

Thermal plantar test was performed to 4 groups at the 4th and 12th weeks after surgery similar as previously described [25]. To assess the nociceptive response to thermal stimuli, the hind paw was immersed into the water bath at 42 °C, then the paw withdrawal latency was recorded 5 times in every detection, which was separated by a minimum interval of 10 min. And paw retractions resulting from locomotion or weight shifting were not included.

### Detection of CAMP and MCV and retrograde axonal tracing with FQ

As previously described, 12 weeks after grafting, compound muscle action potential (CMAP) was measured, and the motor nerve conduction (MCV) was calculated [26]. FG retrograde tracing was performed as previously described [27]. In brief, the sciatic nerve at the injured side was re-exposed under anesthesia. A total volume of 1 mL of 5% fluorogold (FG) (Fluorochrome, Inc., Denver, CO) in sterile saline was injected into the regenerated nerve trunk 5 mm from the distal end, and then the incision was closed. After survival for 5 days, rats were deeply anesthetized and transcardially perfused with saline followed by 4% (v/v) paraformaldehyde in 0.1 M phosphate-buffered saline (pH 7.2). The L4-L6 spinal cord segment and the L4-L6 DRGs were excised and rinsed in the same fixative overnight at 4 °C. After processing with a graded sucrose series, samples were serially sectioned on a cryostat either longitudinally (for DRGs, 10 μm thick) or transversely (for spinal cords, 15 μm thick); 12–16 sections of DRGs and 60–80 sections of spinal cord specimens were harvested respectively. In every group, slices were cross-stained with Hoechst33342. Total fields imaging were taken under a laser scanning confocal microscope (Leica, Wetzlar, Germany) from each section. The FG-retrogradely labeled motor neurons were counted to obtain the total number of FG-labeled motor neurons, and the FG-labeled sensory neurons in the DRGs were calculated as the cell ratio (the percentage of FG-labeled sensory neurons).

### Morphometric analysis of myelinated regenerated nerve

After 12 weeks, implants were excised, fixed, and cut into ultrathin sections, which were post-fixed and stained with lead citrate and uranyl acetate, followed by observation under TEM (Jeol Ltd., Tokyo, Japan) as previously described [27]. And images were taken from 10 random fields of each section to measure the thickness of myelin sheaths, the diameter of myelinated nerve fibers, and the number of myelin sheath layers using Image Pro Plus software (Media Cybernetics, Silver Spring, MD).

### Morphometric analysis of muscle

#### Muscle wet weight ratio

After nerve grafting of 12 weeks, gastrocnemius, anterior tibial muscles, soleus, and extensor digitorum longus on the injured and contralateral, uninjured sides were harvested from deeply anesthetized animals and immediately weighed, respectively, to determine the wet weight ratio of muscles (the wet weight of muscle on the injured side/the wet weight of muscle on the uninjured side).

#### Masson trichrome staining

The gastrocnemius and anterior tibial muscle was harvested from the mid-belly of the injured and contralateral uninjured limbs in rats. The muscle samples were prepared into paraffin sections, which were then subjected to Masson trichrome staining as previously described [27]. The contralateral uninjured muscle sample served as normal control.

#### Motor endplate analysis

Other muscle samples were post-fixed and cut on a cryostat into longitudinal sections; acetylcholinesterase (AChE) histochemical staining was applied to the gastrocnemius and anterior tibial muscle sections followed by observation under light microscopy.

#### TEM observation

The same as regenerated nerves, muscle samples were prepared to be observed under TEM.

### Repair of OGD-injured DRG neurons

The rBM-NCPs and rBM-MSCs in exponential growth phase were cultured in neurobasal medium (Gibco) without FBS for 24 h, then NCP-CM and MSC-CM was collected and cryopreserved at negative 20 °C.

Primary DRG neurons were cultured as previously described [18]. Briefly, DRGs were collected from 6-week-old rats, then digested sequentially in 3 mg/mL collagenase (Sigma) for 30 min and 0.25% trypsin (Sigma) for 30 min. DRGs were mechanically dissociated to achieve single-cell suspension and cultured in DMEM medium supplemented with 5% FBS. Then, the cells were purified by differential velocity adherent technique and treatment with antimitotics: fluorodeoxyuridine (10 mM, Sigma) and uridine (10 mM, Sigma). The cells were replated on PLL-coated dishes at a density of 5 × 10^4^ cells/cm^2^ in neurobasal medium supplemented with 1% B27, 50 ng/mL nerve growth factor (NGF, Sigma), and 2 mM l-glutamine (Gibco) for at least 6 days with medium being refreshed every 2–3 days. Then, neurons were cultured in non-glucose neurobasal medium (Gibco) in 37 °C incubator with 5% CO_2_ and 0.1% oxygen concentration for exposure to 8 h OGD, followed by the treatment with either NCP-CM, MSC-CM, or NM in a 37 °C incubator with 5% CO_2_ and 21% oxygen concentration for 24 h; additionally, PI3K/AKT signal pathway inhibitor LY294002 (Cell Signaling Technology) was added to NCP-CM. The morphology of DRG neurons was observed under phase-contrast microscope. Immunofluorescent staining for β-Tubulin III was analyzed by Leica Qwin V3 image processing software. We assessed three parameters: the percentage of neurons with longest neurite more than double diameter of soma, the average length of the longest neurite, and the ratio of neurite density to cell numbers (number of branches per hundred neurons).

### qRT-PCR

Total RNA was extracted from the cells, and cDNA was synthesized with an Omniscript RT kit (Qiagen, Valencia, CA). Real-time PCR analysis was performed to assay the relative expression of genes in rBM-NCPs and rBM-MSCs (^△△^Ct method). The mRNA expression of secreted bioactive factors were determined, including EGF, platelet-derived growth factor alpha (PDGFα), hepatocyte growth factor (HGF), glial-cell-line-derived neurotrophic factor (GDNF), NGF, ciliary neurotrophic factor (CNTF), vascular endothelial growth factor alpha (VEGFα), and angiogenin (ANG). The primer sequences for each gene are described in Additional file 1: Table S1.

### Statistical analysis

All quantitative data were presented as means ± SE. Comparison between groups was assessed by the Student *t* test, and *p* < 0.05 was considered statistically significant. Curve graphs analysis between each other was assessed by the Kolmogorov-Smirnov test. Statistical analysis was conducted using GraphPad Prism 6.0 software.

## Results

### Characterization and tracking of rBM-NCPs

**Fig. 1:**
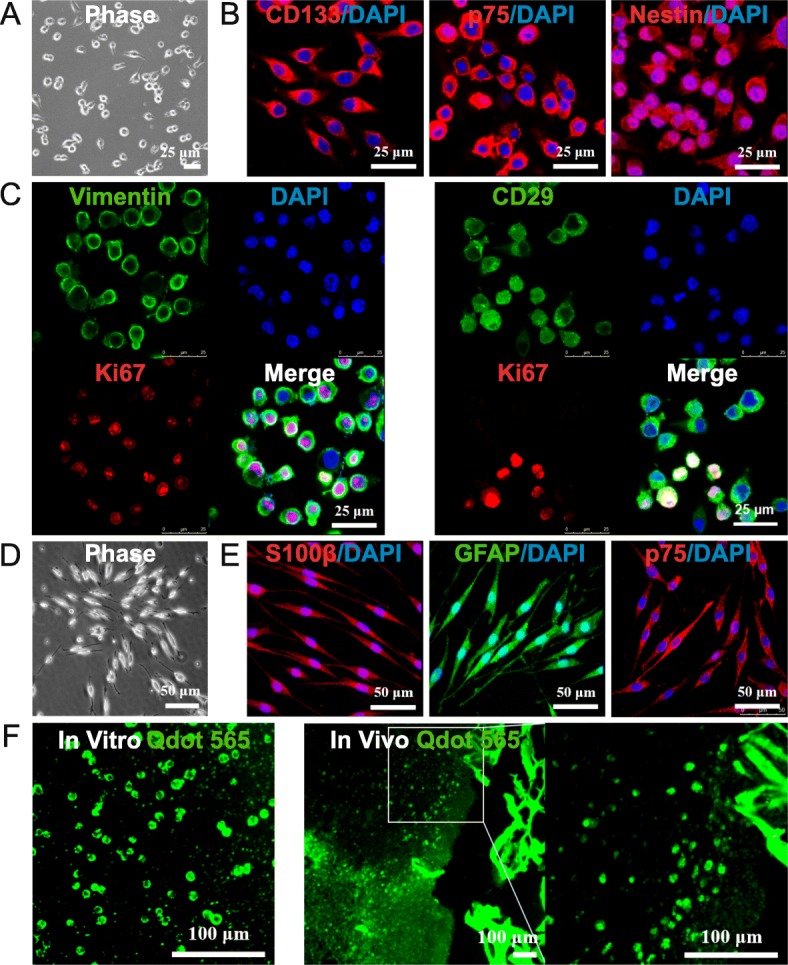
Characterization and tracking of rBM-NCPs. **a** The rBM-NCPs in adherent monolayer culture on PLL-coated plates showed round or short-spindle shape. **b** Immunofluorescent staining of rBM-NCPs demonstrated positive expression of neural crest markers CD133 (red), p75 (red), and Nestin (red), and cell nuclei were labeled with DAPI (blue). **c** Immunofluorescent staining of rBM-NCPs demonstrated positive expression of neural crest markers Vimentin (green in left panel) or CD29 (green in right panel) with proliferation marker Ki67 (red) and DAPI (blue) labeled cell nuclei. **d** Induced Schwann cells from differentiated rBM-NCPs showed spindle-like shape with a side-by-side alignment. **e** Induced Schwann cells demonstrated positive expression of Schwann cell markers S100β (red), GFAP (green), and p75(red), and cell nuclei were labeled with DAPI (blue). **f** The rBM-NCPs in adherent monolayer culture were labeled with Qdot-tracker 565 (green) in vitro (left panel) and detectable in frozen sections of TENG after transplantation for 1 week with local magnification (right panel). Scale bars, 25 μm (**a**, **b**, **c**), 50 μm (**d**, **e**), and 100 μm (**f**)

The rBM-NCPs attached on PLL-coated culture ware showed round or short-spindle shape (Fig. 1a). Immunofluorescence analysis confirmed the positive expression of neural crest markers CD133, p75, and nestin (Fig. 1b), and the co-expression of proliferation marker Ki67 with neural crest marker CD29 or vimentin by rBM-NCPs. It suggested that monolayer-cultured rBM-NCPs could sustain the proliferative capacity and NCP phenotype (Fig. 1c).

The induction of differentiation displayed that the derived Schwann cells from rBM-NCPs became elongated long-spindle shape and side-by-side alignment (Fig. 1d), as well as positive expression of Schwann cell-specific markers, including S100β, GFAP and p75 (Fig. 1e).

After the characterization of the monolayer-cultured rBM-NCPs, cells were labeled with Qdot-tracker 565 and detected immediately in vitro, and the labeled cells were still detectable in frozen sections of TENG after transplantation for 1 week (Fig. 1f).

### Hind limb motor and sensory function scores

**Fig. 2:**
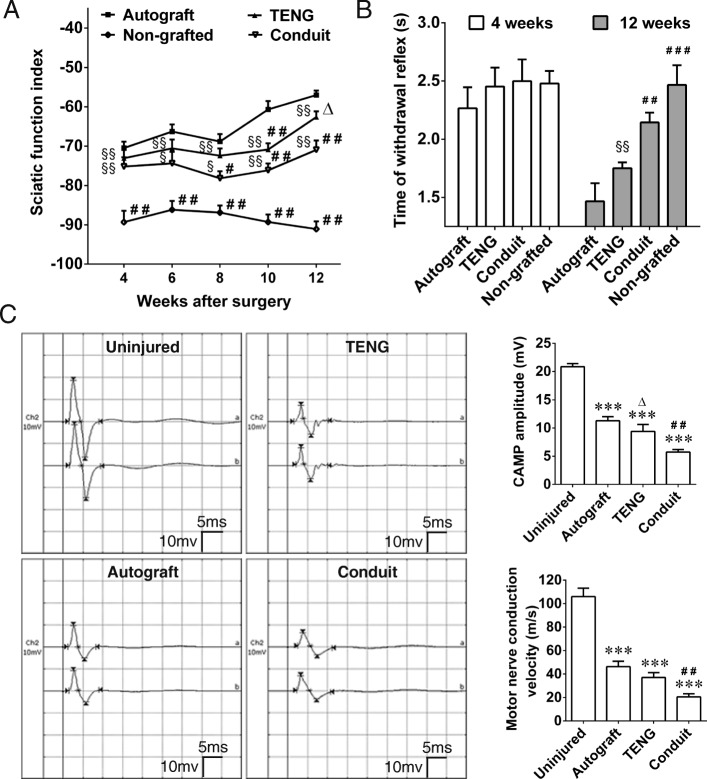
Functional evaluations of rat sciatic nerve after bridging defect with TENG. **a** Curve graph showing the SFI value, the motor function scores of rat hind limb, in 3 grafted groups and the non-grafted group at indicated time points within 12 weeks, and the non-grafted group was set as the control group. **b** Histogram showing the sensory function scores, the withdrawal response time of rat paw in the autograft, TENG, conduit, and non-grafted groups was all delayed at the 4th week and was reduced to different degree at the 12th week. **c** Representative CMAP recording image at 12th week after grafting by autograft, TENG, or conduit, and the contralateral uninjured side was set as the control group. Histograms showing CMAP amplitude and MCV evaluation at 12th week after grafting. *n* = 8; ***p < 0.001 compared with the uninjured group; ^#^*p* < 0.05, ^##^*p* < 0.01, ^###^*p* < 0.001 compared with the autograft group; ^△^*p* < 0.05 compared with the conduit group; ^§^*p* < 0.05, ^§§^*p* < 0.01 compared with the non-grafted group

We assessed the repair potential of rBM-NCPs served as seeding cells of TENG after injection into the conduits to bridge 10 mm sciatic nerve defects in rats. Walking track analysis was performed to evaluate the motor activity of rat hind limb. The SFI value was zero (normal) before surgery and decreased dramatically to negative 100 immediately after surgery (complete dysfunction). Afterwards, the SFI value in each group was turned from the increase tendency (from 4 weeks to 6 weeks after grafting) to the decrease tendency (from 6 weeks to 8 weeks after grafting). Notably, the SFI value in 3 grafted groups showed increase tendency again from 8 to 12 weeks after grafting. Collectively, at 12 weeks after grafting, there was no statistical difference between the TENG group and the autograft group, and the SFI value was higher in the TENG group than that in the conduit group and non-grafted group (Fig. 2a).

The recovery of sensory function was evaluated by observation of the response of rat paw to thermal stimulation. The time of retraction response was measured in 4 groups. After nerve injury, the time of withdrawal reflex of all animals was delayed dramatically at 4 weeks after grafting. Collectively, at 12 weeks after grafting, the withdrawal reflex time of animals in 3 grafted groups was markedly shortened than that in the non-grafted group, and there was no statistical difference between the TENG group and the autograft group, and animals in the TENG group showed a more significant recovery than that in the conduit group in spite of no statistical difference (Fig. 2b).

At 12 weeks after grafting, the value of CMAP and motor nerve conduction MCV detected at the injured side was significantly less than that at the contralateral uninjured side in each grafted group, and no CMAP value was recorded at the injured side in the non-grafted group. The CMAP amplitude and the MCV value were significantly better in the TENG group than those in the conduit group, although the best value was recorded in the autograft group (Fig. 2c).

**Fig. 3:**
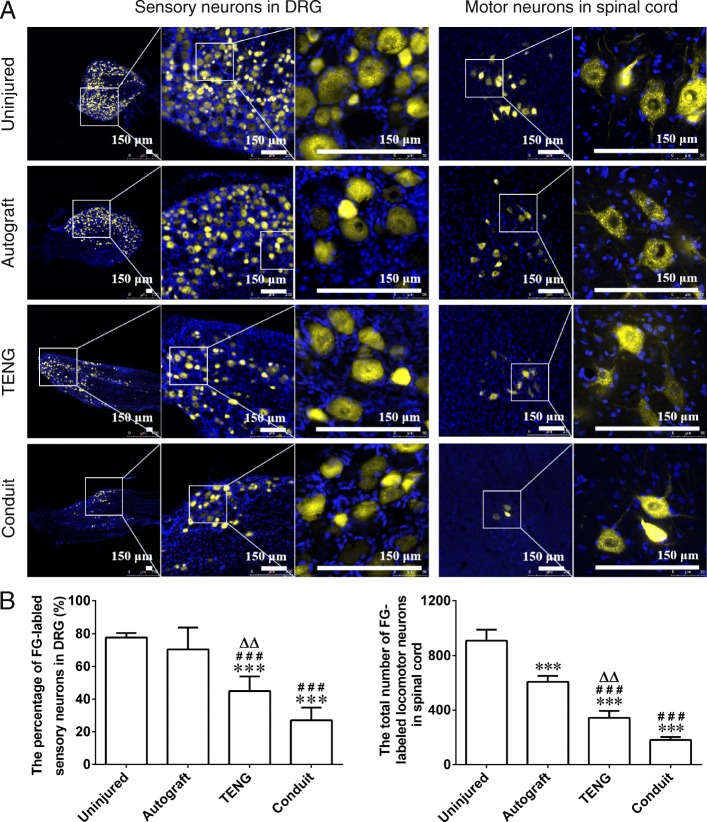
Retrograde axonal tracing with FG in sensory neurons or motor neurons. **a** Representative image of FG-labeled sensory neurons in DRGs or motor neurons in the anterior horn of gray matter of spinal cord in the autograft, TENG, conduit, and uninjured groups at the 12th week after grafting; the golden-colored fluorescence was concentrated in the cell body of neurons. Scale bar, 150 μm. **b** Histograms showing the percentage of FG-labeled sensory neurons and the total number of FG-labeled motor neurons in 4 groups. *n* = 4; ***p < 0.001 compared with the uninjured group, ^###^*p* < 0.001 compared with the autograft group, ^△△^*p* < 0.01 compared with the conduit group

Retrograde axonal tracing with FG revealed that FG-labeled sensory neurons appeared at DRGs and FG-labeled motor neurons appeared at the anterior horn of gray matter in spinal cord in 3 grafted groups at 12 weeks after grafting. The golden-colored fluorescence was concentrated in the cell body of neurons (Fig. 3a). The percentage of FG-labeled sensory neurons or the total number of FG-labeled motor neurons in the TENG group was significantly more than that in the conduit group, although was less than that in the autograft group or uninjured group (Fig. 3b).

### TEM observation of regenerated nerves

**Fig. 4:**
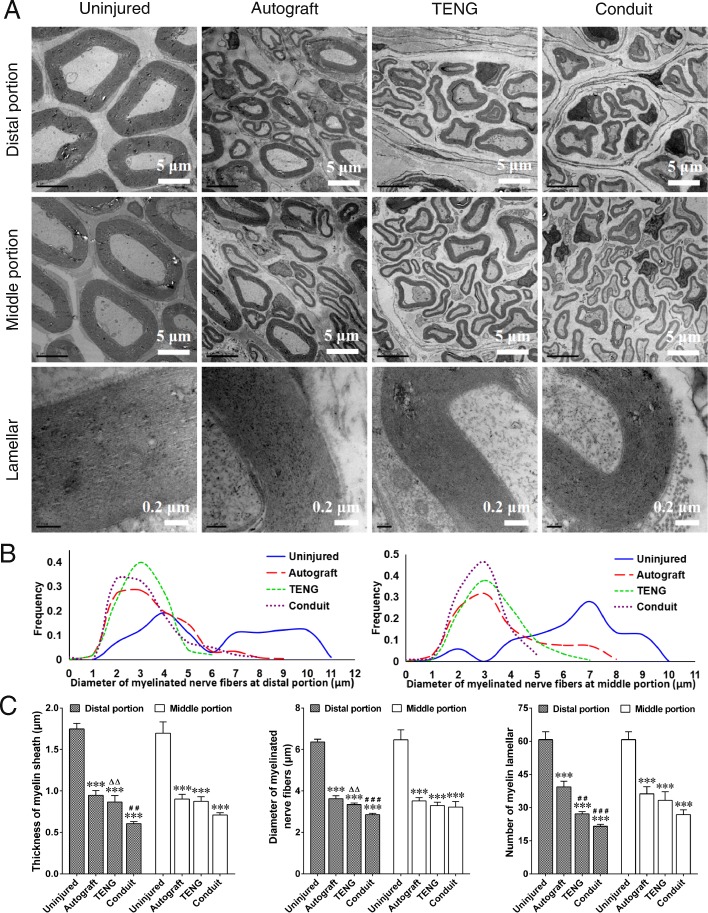
TEM observation of regenerated nerve fibers. **a** Representative TEM image showing that the regenerated nerve fibers dispersed in clusters at both the distal portion and the middle portion in autograft, TENG, and conduit groups, and the myelinated axons were surrounded by clear, thick, electron-dense myelin sheaths with lamellar structure, and the contralateral uninjured side was set as the control group. Scale bars, 5 μm (distal portion and middle portion), and 0.2 μm (lamellar). **b** Curve graphs of the diameter distribution of the myelin sheaths showing a peak value at about 3 μm at both the distal and the middle portion of the regenerated nerve fibers in 3 grafted groups, and displayed significant differences between each other in terms of the Kolmogorov-Smirnov test, *n* = 4. **c** Histograms showing the thickness, diameter, and myelin lamellar number of myelin sheath at the distal and middle portion of regenerated nerve fibers in 3 grafted groups, and the contralateral uninjured side was set as the control group. *n* = 4; ***p < 0.001 compared with the uninjured group; ^##^*p* < 0.01, ^###^*p* < 0.001 compared with the autograft group; ^△△^*p* < 0.01 compared with the conduit group

In the 3 grafted groups, TEM observation confirmed the regeneration of myelinated nerve fibers, which appeared as massive bundles at distal portion and middle portion of the regenerated nerve. The myelinated fibers were dispersed in clusters in the regenerated nerve, and the myelinated axons were surrounded by clear, thick, electron-dense myelin sheaths with lamellar structure, although the myelin sheaths in 3 grafted groups were thinner than that in the contralateral, uninjured group. And the myelin lamellar structure provided further evidence for the regeneration of injured nerve (Fig. 4a).

Image analyses showed that the diameter distribution curves of the myelin sheaths reached a peak value at about 3 μm at both the distal and middle portion of the regenerated nerve fibers in 3 grafted groups, which displayed significant differences between the uninjured group and grafted groups in terms of the Kolmogorov-Smirnov statistical test (Fig. 4b). Moreover, image analysis of the distal portion of regenerated nerve fibers demonstrated that the thickness, diameter, and myelin lamellar number of myelin sheath in the TENG group and the autograft group were greater than those in the conduit group, although the value was less than that in the uninjured group. Furthermore, the thickness and diameter of myelin sheath in the TENG group were equal to those in the autograft group, even though the myelin lamellar number in the TENG group was less than that in the autograft group. And image analysis of the middle portion of regenerated nerve fibers showed no statistical difference among 3 grafted groups (Fig. 4c).

### Histology and morphology observation of hind limb muscles

**Fig. 5:**
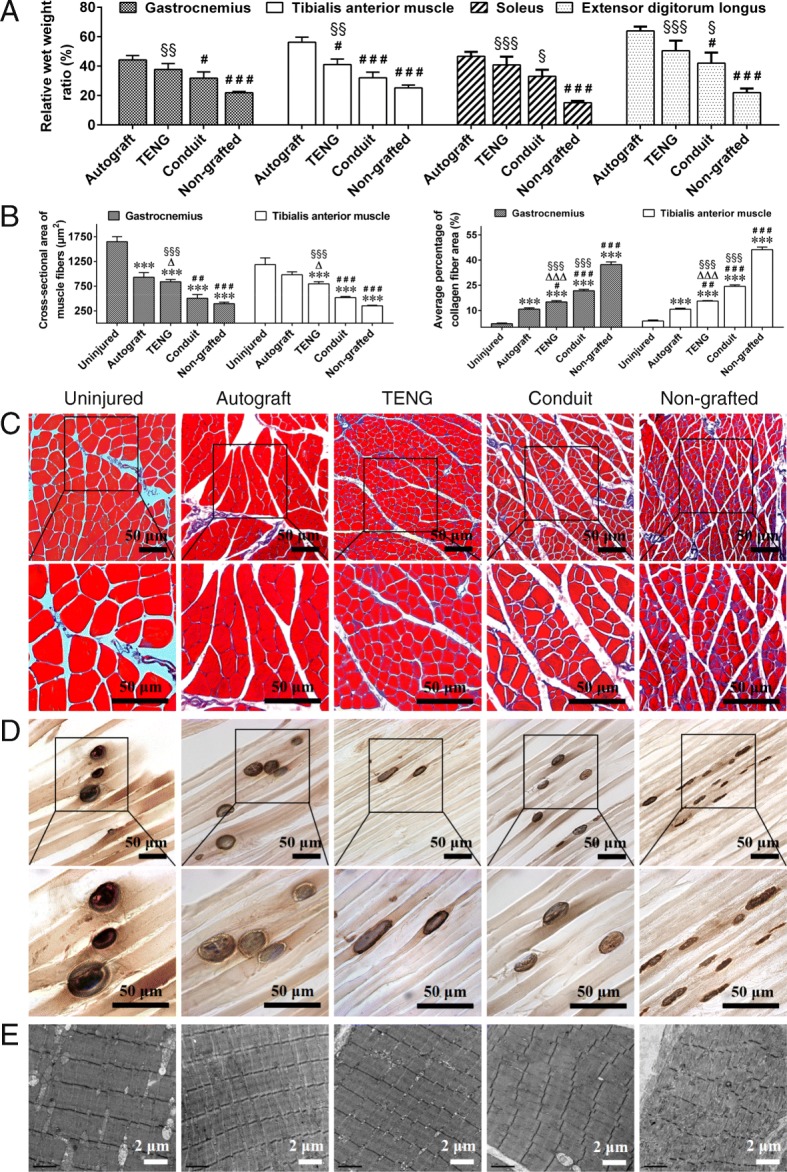
Histology and morphology observation of hindlimb muscles. **a** Histograms showing the difference of wet weight ratio of targeted muscles (injured side/contralateral uninjured side) among the non-grafted group and 3 grafted groups. *n* = 8; ^#^*p* < 0.05, ^###^*p* < 0.001 compared with the autograft group, ^§^*p* < 0.05, ^§§^*p* < 0.01, ^§§§^*p* < 0.001 compared with the non-grafted group. **b** Histograms showing the cross-sectional area of muscle fiber and the average percentage of collagen fiber area of gastrocnemius muscle and tibialis anterior muscle in uninjured, autograft, TENG, conduit, and non-grafted groups (see also Additional file 2: Figure S1A). *n* = 4; ***p < 001 compared with the uninjured group; ^#^*p* < 0.05, ^##^*p* < 0.01, ^###^*p* < 0.001 compared with the autograft group; ^△^*p* < 0.05, ^△△△^*p* < 0.001 compared with the conduit group; ^§§§^*p* < 0.001 compared with the non-grafted group. **c** Representative images of Masson trichrome staining of cross-sectional gastrocnemius muscle in uninjured, autograft, TENG, conduit, and non-grafted groups. Scale bar, 50 μm. **d** Representative images of cholinesterase histochemistry staining for motor endplates of longitudinal gastrocnemius muscle section in uninjured, autograft, TENG, conduit, and non-grafted groups. Scale bar, 50 μm. **e** Representative TEM images of muscle segment morphology of gastrocnemius muscle in uninjured, autograft, TENG, conduit, and non-grafted groups. Scale bar, 2 μm

At 12 weeks after grafting, the wet weight ratio of targeted muscles (injured side/contralateral uninjured side) showed significant difference among the non-grafted group and 3 grafted groups (Fig. 5a). Masson trichrome staining of gastrocnemius muscle and tibialis anterior muscle was analyzed, which showed that the cross-sectional area of muscle fiber increased and the average percentage of collagen fiber area decreased in 3 grafted groups (Fig. 5b, c and Additional file 2: Figure S1A). Additionally, AChE histochemistry staining for motor endplates (Fig. 5d and Additional file 2: Figure S1B) and TEM observation (Fig. 5e and Additional file 2: Figure S1C) indicated that the muscle atrophy in 3 grafted groups was less pronounced than that in the non-grafted group, and the alleviation in the TENG group was more pronounced than that in the conduit group, although the alleviation in the autograft group was the greatest. It was demonstrated that the denervation of target muscles and muscle atrophy which resulted from sciatic nerve defects could be attenuated by TENG transplantation.

### Repair effect of rBM-NCPs on injured DRG neurons in vitro

**Fig. 6:**
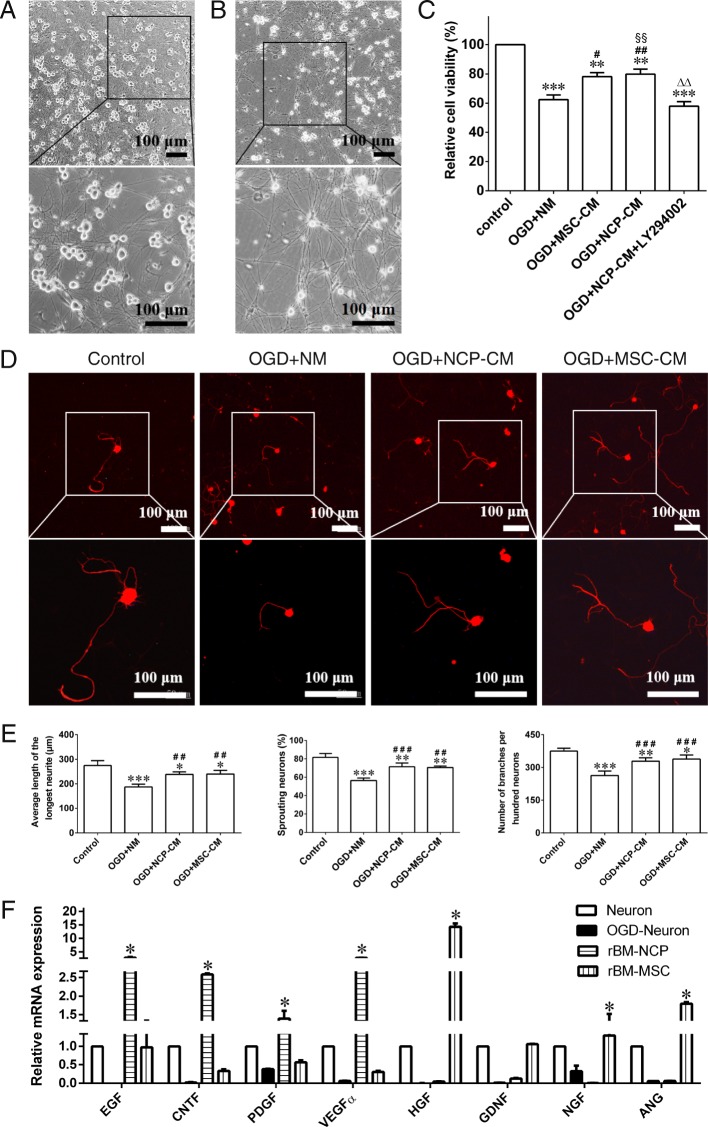
Repair effect of rBM-NCPs on adult rat DRG neurons on exposure to OGD in vitro*.* **a** Phase-contrast image of neurons 6 days after culture. Scale bar, 100 μm. **b** Phase-contrast image showing neurons with disrupted neurites and shrinking neuron soma 8 h after OGD. Scale bar, 100 μm. **c** Histogram showing the relative cell viability of neurons. OGD damaged neurons 24 h after treatment with NCP-CM showed significantly increased viability compared with treatment with NM and showed equal viability compared with treatment with MSC-CM. And the increased viability of treated neurons could be inhibited by LY294002 (a blocking agent of PI3K/AKT pathway). *n* = 3; **p < 0.01, ***p < 0.001 compared with control group; ^#^*p* < 0.05, ^##^*p* < 0.01 compared with OGD+NM group; ^△△^*p* < 0.01 compared with OGD+MSC-CM group; ^§§^*p* < 0.01 compared with OGD+NCP-CM+LY294002 group. **d** Representative image showing positive expression of neural marker β-tubulin III (red) in soma and sprouting neurites of neurons in different groups. Scale bar, 100 μm. **e** Histograms showing the average length of the longest neurites, the ratio of neurites density to cell numbers, and the percentage of sprouting neurons with longest neurite more than double diameter of soma of neurons in different groups. *n* = 3; *p < 0.05, **p < 0.01, ***p < 0.001 compared with control group; ^##^*p* < 0.01, ^###^*p* < 0.001 compared with OGD+NM group. **f** Histograms showing the relative mRNA expression of trophic factors for axonal regeneration and angiogenesis by qRT-PCR analysis in normal neurons, OGD injured neurons, rBM-NCPs and rBM-MSCs, indicated EGF, CNTF, PDGFα, and VEGFα genes were higherly expressed in rBM-NCPs, as well as HGF, GDNF, NGF, and ANG genes were higherly expressed in rBM-MSCs*. n* = 3; *p < 0.05, **p < 0.01, ***p < 0.001 compared with normal neuron

In order to further examine the repair effect of rBM-NCPs on the injured neurons, MSCs of rat bone marrow origin (rBM-MSCs) were set as positive control. We expanded rBM-MSC cell line clone 16 that had been established in our previous work [19]. Then, NCP-CM and MSC-conditioned medium (MSC-CM) was collected. Compared with neurons from DRGs of adult rats cultured in normal medium (NM) (Fig. 6a), the injured neurons showed disrupted neurites and shrinking neuron soma at 8 h after exposure to OGD (Fig. 6b). At 24 h after treatment with NCP-CM, MSC-CM, or NM, OGD-neurons in the NCP-CM group showed significantly increased viability compared with that in the NM group and showed no significant difference with that in the MSC-CM group. And the increased viability of NCP-CM treated OGD-neurons could be inhibited by LY294002 that is the blocking agent of phosphatidylinositol-3-kinase/serine-threonine kinases (PI3K/AKT) pathway (Fig. 6c).

Moreover, the immunofluorescence staining of β-tubulin III for neurons was further analyzed by Leica Qwin V3 image processing software (Fig. 6d). At 24 h after treatment, comparing with OGD-neurons treated with NM, OGD-neurons treated with NCP-CM or MSC-CM displayed longer average length of the longest neurites, increased ratio of neurites density to cell numbers, as well as percentage of neurons, with longest neurite more than double diameter of soma, also increased (Fig. 6e).

The rBM-NCPs have been identified as SCPs in our previous work, it is possible that the transplanted SCPs may provide trophic factors that stimulate the regeneration after nerve injury. Real-time qPCR analysis confirmed that neurotrophic factors, such as EGF, CNTF, PDGFα, and VEGFα genes showed higher expression level in rBM-NCPs than in normal neurons, injured neurons and rBM-MSCs, and HGF, GDNF, NGF and ANG genes were higher expressed in rBM-MSCs than in normal neurons, injured neurons and rBM-NCPs (Fig. 6f). The results suggested that rBM-NCPs and rBM-MSCs may provide various trophic factors for axonal regeneration and angiogenesis.

## Discussion

Neural crest stem cells (NCSCs) and NCPs are initially generated from neural crest and migrate throughout the body to produce a diverse array of mature tissue types. Emerging evidence suggests that the postmigratory neural crest cells may reside in the postnatal tissues such as dental pulp [28, 29], hair follicle [30, 31], DRG, and bone marrow [3]. It provides more possibility to obtain neural crest-derived cells for repair of long-distance peripheral nerve defects or gross nerve damage. Our results indicated that the constructed TENG with rBM-NCPs loaded into conduits showed repair effectiveness superior to the conduits alone in bridging the 10-mm distance sciatic nerve gap in rats, which was a developing work of rBM-NCPs on facilitating nerve regeneration. This work suggested the repair potential of rBM-NCPs to long-distance nerve defects besides nerve crush injury. The transplantation of TENG promoted the reinnervation of hindlimb muscle and skin that enhanced the rehabilitation of motor and sensory function of rats within 12 weeks. Moreover, based on our findings of the restoring of OGD injured neurons with NCP-CM in vitro, we deduced that multiple trophic factors in the secretome of rBM-NCPs could contribute to the axonal regeneration and functional recovery of neurons in the ischemic microenvironment in vivo.

Here rBM-NCP cell subpopulation is available SCPs with rational source and negligible damage to organism; rBM-NCPs implantation did not appear immunological rejection and demonstrated the tissue compatibility with chitosan and silk fibroin fiber. After transplantation, the seeding rBM-NCPs survived in conduits, and TENG transplantation obtained higher repair effect than conduits alone. Recent evidence illustrated that the selection of appropriate stem cells and their derivatives for cell-based therapy is critical in nerve regeneration, and iPSC-derived NCSCs promoted better motor nerve recovery and long-term muscle recovery than mature Schwann cells derived from iPSC-NCSCs [32]. Accordingly, rBM-NCPs were selected as seeding cells rather than rBM-NCPs-derived Schwann cells. Additionally, the same as rBM-MSCs, rBM-NCPs can avoid either ethical problem or gene conversion problem of embryonic stem cell (ESC)-derived or iPSC-derived neural crest cells. These desirable features are favorable for rBM-NCPs to be the novel candidates to enhance the repair of peripheral nerve defects based on cell therapy.

Even though diverse seeding cells were applied in the reported trial of peripheral nerve repair, how to maintain abundant, viable, and potential cells in a steady state has been a serious obstacle to cell transplantation. In this work, the adherent monolayer-cultured rBM-NCPs sustained proliferating and avoided the undesirable cell viability inside the floating spheres. Similar to the reported results from neural stem/precursor cells for neural tissue engineering applications [33], monolayer-cultured rBM-NCPs showed anticipated NCC state with positive expression of neural crest marker CD133, p75, nestin, vimentin, and CD29, as well as cell proliferation marker Ki67. Moreover, the induced Schwann cells from monolayer-cultured rBM-NCPs were characterized with positive expression of S100β, GFAP, and p75, which demonstrated the stable SCP identity of rBM-NCPs. In addition, for tracking in vivo, it is imaginable that the in vitro monolayer-cultured rBM-NCPs could be labeled more effectively than rBM-NCPs spheres. Our findings showed the survival of Qdot-nanocrystal-565-conjugate-labeled rBM-NCPs in TENG tracked 7 days after transplantation. Hence, in our future work, it would be desirable to apply more effective tracer to realize the tracking of transplanted cells for longer time to assess cell behavior at more time points in regeneration process. It would be beneficial to further inspect the survival, migration, contribution, and fate of the engrafted cells in peripheral nerve regeneration process.

To date, in order to restore sensory and motor function of rat sciatic nerve with long-distance defects, there are several problems that remain to be elucidated such as orderly nerve fiber regeneration, neurite fasciculation, accurate reinnervation of motor end plates, and remodeling of synapse structures of sensory and motor neurons. These issues are closely associated with the nerve regeneration microenvironment. In the creation of peripheral nerve regeneration microenvironment, one of the main encountered obstacles is the lack of enough endogenous repair type Schwann cells transformed from myelin and nonmyelin (Remak) Schwann cells [34]. Accordingly, the transplantation of seeding cells, such as cultured primary Schwann cells, iPSC-derived Schwann cells, or adult tissue-derived MSCs, might took advantage of their respective features to provoke and prompt the response of endogenous Schwann cells to nerve injury. Especially, it was reported that the engraftable MSCs tended to exert a paracrine effect on surrounding microenvironment and could stimulate the injured axon to regenerate [35], and the endogenous repair type Schwann cells would eventually transform into myelinating Schwann cells wrapping around the regenerated nerve [36].

In present study, our results of the assessment of behavior, electrophysiology, and retrograde tracing demonstrated that rBM-NCPs enhanced the survival and functional recovery of sensory and motor neurons via. Additionally, we showed the regrowth and remyelination of damaged axons and the reversing of the atrophy of denervated muscles via histology and morphology observation even by TEM. It is noteworthy that the repair effect of rBM-NCPs was consistent with the repair effect of neural crest stem-like cells induced from human gingiva-derived MSCs and dental pulp stem cells on peripheral nerve injury [28, 37]. Abovementioned cell subpopulations might confer similar properties of NCCs despite their different tissue source and play an equal role in peripheral nerve regeneration. Most recently, it was reported that human ESC-derived NCCs are biologically active and may provide trophic support to stimulate peripheral nerve regeneration [38], and implantation of iPSC-derived NCCs may even improve enteric nervous system function [39]. Considering the strategies of stem cell-based therapy on the augmentation of peripheral nerve regeneration, current opinions mostly emphasize on the secretion of bioactive factors that orchestrate signaling transfection to conceive the microenvironment necessary for nerve regeneration, far from being mere spare parts for cell replacement or supplement therapy [40, 41].

Moreover, we verified the repair effect of NCP-CM on injured primary sensory neurons cultured from adult rat DRGs in vitro and further compared some trophic factors generated by rBM-NCP subpopulation with previously established rBM-MSC subpopulation. Those trophic factors, such as EGF, PDGFα, CNTF, VEGFα, HGF, GDNF, and NGF, are signal molecules of PI3K/Akt pathway, that is an upstream signal of both Glycogen synthase kinase 3-β (GSK3-β) and cytoskeletal arrangements for axon extension [42]. The trophic factor activity is closely relevant with both neuronal survival and axonal regrowth, even implicated with angiogenesis and remyelination [43–46]. Previous investigations have revealed that angiogenesis should be one essential prerequisite for encouraging neurogenesis in peripheral nerve regeneration [47]. It is implied that rBM-NCPs can exert a target-derived trophic support to both neuronal survival and axonal regrowth through secreted cytokines.

Intriguingly, our findings displayed different mRNA expression profile of higher level trophic factors between rBM-NCPs and rBM-MSCs, which might correlate with their distinct cell lineage type and biological function. This result is illuminating, and the further comparison of the secretome between rBM-NCPs and rBM-MSCs may be accomplished by protein array technologies for proteomics. Additionally, further exploring the paracrine action mechanism of implanted cells, the bioactive factors released from donor cells may be delivered as messages to recipient cells, including soluble active factors and extracellular vesicle (EV). Bioactive factors including diverse biochemical substantial style such as nucleic acid and lipid, besides protein peptide, can also be contained in EV. It has been reported that different types of EV would communicate to different cells in variable states [48]. We need further investigation to clarify the intercellular communication, the secretome of transplanted cells, and the responding of surrounding cells, in creating neuroregenerative microenvironment. Selectively or synergistically applying enriched bioactive factors to promote nerve regeneration should be in consideration depending on practical demand.

## Conclusions

Taken together, this work demonstrates the effective reproduction and expansion of rBM-NCPs in adherent monolayer culture and provides an alternative supporting cell source for the construction of TENG. Here rBM-NCPs hold great promise to create the microenvironment for nerve regeneration through secreted bioactive factors. The sciatic nerve defect repair partially depends on the trophic factors, and the secreted bioactive factors from rBM-NCP and rBM-MSC are both beneficial for the restoration of OGD-injured DRG neurons. Moreover, detailed understanding of the secretome of rBM-NCPs differing from rBM-MSCs would be conductive to reveal the underlying molecular mechanisms of implanted cells on improving neuroregeneration and develop a new paradigm for cell-free regeneration.

## Supplementary information

**Additional file 1: Table S1.** Primers used for real-time polymerase chain reaction.

**Additional file 2: Figure S1.** Histological observation of tibialis anterior muscle. **a** Representative images of masson trichrome staining of cross-sectional tibialis anterior muscle in uninjured, autograft, TENG, conduit and non-grafted groups. Scale bar, 50 μm. **b** Representative images of cholinesterase histochemistry staining for motor endplates of longitudinal tibialis anterior muscle section in uninjured, autograft, TENG, conduit and non-grafted groups. Scale bar, 50 μm. **c** Representative TEM images of muscle segment morphology of tibialis anterior muscle in uninjured, autograft, TENG, conduit and non-grafted groups. Scale bar, 2 μm.

## Data Availability

The datasets used and/or analyzed during the current study are available from the corresponding author upon reasonable request.

## Abbreviations

AChE: Acetylcholinesterase
ANG: Angiogenin
CMAP: Compound muscle action potential
CNTF: Ciliary neurotrophic factor
DRG: Dorsal root ganglia
EGF: Epidermal growth factor
ESC: Embryonic stem cell
EV: Extracellular vesicle
FBS: Fetal bovine serum
FG: Fluorogold
FGF2: Fibroblast growth factor-2
GDNF: Glial-cell-line derived neurotrophic factor
GFAP: Glial fibrillary acidic protein
HGF: Hepatocyte growth factor
GSK3-β: Glycogen synthase kinase 3-β
HRG-1β: β-heregulin 1
iPSC: Induced pluripotent stem cell
MCV: Motor nerve conduction
MSC: Mesenchymal stem cell
MSC-CM: MSC-conditioned medium
NCC: Neural crest-derived cell
NCP: Neural crest precursor
NCP-CM: NCP-conditioned medium
NCSC: Neural crest stem cell
NM: Normal medium
NGF: Nerve growth factor
OGD: Oxygen-glucose-deprivation
PDGFα: Platelet-derived growth factor alpha
PI3K/AKT: Phosphatidylinositol-3-kinase/serine-threonine kinases
PLL: Poly-l-lysine
rBM-MSC: Mesenchymal stem cell of bone marrow origin
rBM-NCP: Neural crest precursor of rat bone marrow origin
SCP: Schwann cell precursor
SFI: Sciatic function index
TEM: Transmission electron microscopy
TENG: Tissue engineering nerve graft
tRA: All-trans-retinoic acid
VEGFα: Vascular endothelial growth factor alpha

## References

[1] Jessen KR, Mirsky R (2005). The origin and development of glial cells in peripheral nerves. Nat Rev Neurosci.

[2] Aquino JB (2017). Uncovering the in vivo source of adult neural crest stem cells. Stem Cells Dev.

[3] Nagoshi N, Shibata S, Kubota Y, Nakamura M, Nagai Y, Satoh E (2008). Ontogeny and multipotency of neural crest-derived stem cells in mouse bone marrow, dorsal root ganglia, and whisker pad. Cell Stem Cell.

[4] Aquino JB, Sierra R (2018). Schwann cell precursors in health and disease. Glia..

[5] Carr MJ, Johnston AP (2017). Schwann cells as drivers of tissue repair and regeneration. Curr Opin Neurobiol.

[6] Johnston AP, Yuzwa SA, Carr MJ, Mahmud N, Storer MA, Krause MP (2016). Dedifferentiated Schwann cell precursors secreting paracrine factors are required for regeneration of the mammalian digit tip. Cell Stem Cell.

[7] Johnston AP, Naska S, Jones K, Jinno H, Kaplan DR, Miller FD (2013). Sox2-mediated regulation of adult neural crest precursors and skin repair. Stem Cell Rep..

[8] Cattin AL, Lloyd AC (2016). The multicellular complexity of peripheral nerve regeneration. Curr Opin Neurobiol.

[9] Kang H, Lichtman JW (2013). Motor axon regeneration and muscle reinnervation in young adult and aged animals. J Neurosci.

[10] Painter MW, Brosius Lutz A, Cheng YC, Latremoliere A, Duong K, Miller CM (2014). Diminished Schwann cell repair responses underlie age-associated impaired axonal regeneration. Neuron..

[11] Xue C, S. Y, X. G. Stem cell and peripheral nerve regeneration. Stem cells: basics and clinical translation, Springer Press. 2015:219–46.

[12] Hu N, Wu H, Xue C, Gong Y, Wu J, Xiao Z (2013). Long-term outcome of the repair of 50 mm long median nerve defects in rhesus monkeys with marrow mesenchymal stem cells-containing, chitosan-based tissue engineered nerve grafts. Biomaterials..

[13] Li G, Zhao X, Zhao W, Zhang L, Wang C, Jiang M (2014). Porous chitosan scaffolds with surface micropatterning and inner porosity and their effects on Schwann cells. Biomaterials..

[14] Xue C, Hu N, Gu Y, Yang Y, Liu Y, Liu J (2012). Joint use of a chitosan/PLGA scaffold and MSCs to bridge an extra large gap in dog sciatic nerve. Neurorehabil Neural Repair.

[15] Yao M, Zhou Y, Xue C, Ren H, Wang S, Zhu H (2016). Repair of rat sciatic nerve defects by using allogeneic bone marrow mononuclear cells combined with chitosan/silk fibroin scaffold. Cell Transplant.

[16] Kim HS, Lee J, Lee DY, Kim YD, Kim JY, Lim HJ (2017). Schwann cell precursors from human pluripotent stem cells as a potential therapeutic target for myelin repair. Stem cell Rep.

[17] Petersen J, Adameyko I (2017). Nerve-associated neural crest: peripheral glial cells generate multiple fates in the body. Curr Opin Genet Dev.

[18] Shi H, Gong Y, Qiang L, Li X, Zhang S, Gao J (2016). Derivation of Schwann cell precursors from neural crest cells resident in bone marrow for cell therapy to improve peripheral nerve regeneration. Biomaterials..

[19] Shi H, Zhang T, Qiang L, Man L, Shen Y, Ding F (2013). Mesenspheres of neural crest-derived cells enriched from bone marrow stromal cell subpopulation. Neurosci Lett.

[20] Wang X, Hu W, Cao Y, Yao J, Wu J, Gu X (2005). Dog sciatic nerve regeneration across a 30-mm defect bridged by a chitosan/PGA artificial nerve graft. Brain.

[21] Yang Y, Chen X, Ding F, Zhang P, Liu J, Gu X (2007). Biocompatibility evaluation of silk fibroin with peripheral nerve tissues and cells in vitro. Biomaterials..

[22] Yang YM, Yuan XL, Ding F, Yao DB, Gu Y, Liu J (2011). Repair of rat sciatic nerve gap by a silk fibroin-based scaffold added with bone marrow mesenchymal stem cells. Tissue Eng Pt A.

[23] Liu M, Zhang DL, Shao CX, Liu M, Ding F, Gu XS (2007). Expression pattern of myostatin in gastrocnemius muscle of rats after sciatic nerve crush injury. Muscle Nerve.

[24] Bain JR, Mackinnon SE, Hunter DA (1989). Functional evaluation of complete sciatic, peroneal, and posterior tibial nerve lesions in the rat. Plast Reconstr Surg.

[25] Okawa T, Kamiya H, Himeno T, Kato J, Seino Y, Fujiya A (2013). Transplantation of neural crest-like cells derived from induced pluripotent stem cells improves diabetic polyneuropathy in mice. Cell Transplant.

[26] Tang X, Xue CB, Wang YX, Ding F, Yang YM, Gu XS (2012). Bridging peripheral nerve defects with a tissue engineered nerve graft composed of an in vitro cultured nerve equivalent and a silk fibroin-based scaffold. Biomaterials..

[27] Yang Y, Ding F, Wu H, Hu W, Liu W, Liu H (2007). Development and evaluation of silk fibroin-based nerve grafts used for peripheral nerve regeneration. Biomaterials..

[28] Luo L, He Y, Wang X, Key B, Lee BH, Li H (2018). Potential roles of dental pulp stem cells in neural regeneration and repair. Stem Cells Int.

[29] Mead B, Logan A, Berry M, Leadbeater W, Scheven BA (2017). Concise review: dental pulp stem cells: a novel cell therapy for retinal and central nervous system repair. Stem Cells.

[30] Kosykh A, Beilin A, Sukhinich K, Vorotelyak E (2018). Postnatal neural crest stem cells from hair follicle interact with nerve tissue in vitro and in vivo. Tissue Cell.

[31] Yang R, Xu X (2016). Isolation and culture of neural crest stem cells from human hair follicles. Methods Mol Biol.

[32] Huang CW, Huang WC, Qiu X, Fernandes Ferreira da Silva F, Wang A, Patel S (2017). The differentiation stage of transplanted stem cells modulates nerve regeneration. Sci Rep.

[33] Li YC, Tsai LK, Wang JH, Young TH (2014). A neural stem/precursor cell monolayer for neural tissue engineering. Biomaterials..

[34] Jessen KR, Mirsky R, Lloyd AC (2015). Schwann cells: development and role in nerve repair. Cold Spring Harb Perspect Biol.

[35] Sayad Fathi S, Zaminy A (2017). Stem cell therapy for nerve injury. World J Stem Cells.

[36] Gomez-Sanchez JA, Pilch KS, van der Lans M, Fazal SV, Benito C, Wagstaff LJ (2017). After nerve injury, lineage tracing shows that myelin and Remak Schwann cells elongate extensively and branch to form repair Schwann cells, which shorten radically on remyelination. J Neurosci.

[37] Zhang Q, Nguyen PD, Shi S, Burrell JC, Xu Q, Cullen KD (2018). Neural crest stem-like cells non-genetically induced from human gingiva-derived mesenchymal stem cells promote facial nerve regeneration in rats. Mol Neurobiol.

[38] Jones I, Novikova LN, Novikov LN, Renardy M, Ullrich A, Wiberg M (2018). Regenerative effects of human embryonic stem cell-derived neural crest cells for treatment of peripheral nerve injury. J Tissue Eng Regen Med.

[39] Schlieve CR, Fowler KL, Thornton M, Huang S, Hajjali I, Hou X (2017). Neural crest cell implantation restores enteric nervous system function and alters the gastrointestinal transcriptome in human tissue-engineered small intestine. Stem Cell Rep.

[40] Fairbairn NG, Meppelink AM, Ng-Glazier J, Randolph MA, Winograd JM (2015). Augmenting peripheral nerve regeneration using stem cells: a review of current opinion. World J Stem Cells..

[41] Faroni A, Mobasseri SA, Kingham PJ, Reid AJ (2015). Peripheral nerve regeneration: experimental strategies and future perspectives. Adv Drug Deliv Rev.

[42] Chen ZL, Yu WM, Strickland S (2007). Peripheral regeneration. Annu Rev Neurosci.

[43] Funa K, Sasahara M (2014). The roles of PDGF in development and during neurogenesis in the normal and diseased nervous system. J Neuroimmune Pharmacol.

[44] Liu H, Liu G, Bi Y (2014). CNTF regulates neurite outgrowth and neuronal migration through JAK2/STAT3 and PI3K/Akt signaling pathways of DRG explants with gp120-induced neurotoxicity in vitro. Neurosci Lett.

[45] Nowacka MM, Obuchowicz E (2012). Vascular endothelial growth factor (VEGF) and its role in the central nervous system: a new element in the neurotrophic hypothesis of antidepressant drug action. Neuropeptides..

[46] Ojeda L, Gao J, Hooten KG, Wang E, Thonhoff JR, Dunn TJ (2011). Critical role of PI3K/Akt/GSK3beta in motoneuron specification from human neural stem cells in response to FGF2 and EGF. PLoS One.

[47] Cattin AL, Burden JJ, Van Emmenis L, Mackenzie FE, Hoving JJ, Garcia Calavia N (2015). Macrophage-induced blood vessels guide Schwann cell-mediated regeneration of peripheral nerves. Cell..

[48] van Niel G, D'Angelo G, Raposo G (2018). Shedding light on the cell biology of extracellular vesicles. Nat Rev Mol Cell Biol.

