# Supplementary material for: Bone marrow-derived neural crest precursors improve nerve defect repair partially through secreted trophic factors
Source: Stem Cell Res Ther. 2019 Dec 18;10:397. doi: 10.1186/s13287-019-1517-1 (PMC6921427; doi:10.1186/s13287-019-1517-1)
Supplement: Supplementary file 1 — Additional file 1: Table S1. Primers used for real-time polymerase chain reaction. [file 13287_2019_1517_MOESM1_ESM.doc]

**Table S1 Primer sequences for qRT-PCR**

| Gene name | Protein name | Forward Sequence  (5' to 3') | Reverse Sequence  (5' to 3') |
| --- | --- | --- | --- |
| *gapdh* | GAPDH | AAGTTCAACGGCACAGTCAAG | CCAGTAGACTCCACGACATACTCA |
| *egf* | EGF | TTCCCGTGTTCTTCTGAGTTCCTTA | CCTCCAGCAGTGTTTTTACATCCAT |
| *cntf* | CNGF | GTCTAAAGTGTTCCAAAATTGATGC | ATAAGCAGATGTAGCCATTCGC |
| *pdgfa* | PDGFα | CCCACATCGGCCAACTTCTT | TAACCTCACCTGGACCTCTTTCA |
| *vegfa* | VEGFα | CTGTGTGCCCCTAATGCGGT | CTGGCTTTGGTGAGGTTTGATC |
| *hgf* | HGF | CTCTTGACCCTGACACCCCT | ACCTTCTCCTTGGCCTTTTATACAT |
| *gdnf* | GDNF | CTACGAAACCAAGGAGGAACTGA | GGTAAACCAGGCTGTCGTCTAAA |
| *ngf* | NGF | TTTGAGACCAAGTGCCGAGC | CACACACACGCAGGCTGTATCTAT |
| *ang* | ANG | AAGGGTCGGGATGCCAGATACT | CCGTAAGGGCTTCCATTCGC |
