# Supplementary figures and images for: Bone marrow-derived neural crest precursors improve nerve defect repair partially through secreted trophic factors
Source: Stem Cell Res Ther. 2019 Dec 18;10:397. doi: 10.1186/s13287-019-1517-1 (PMC6921427; doi:10.1186/s13287-019-1517-1)

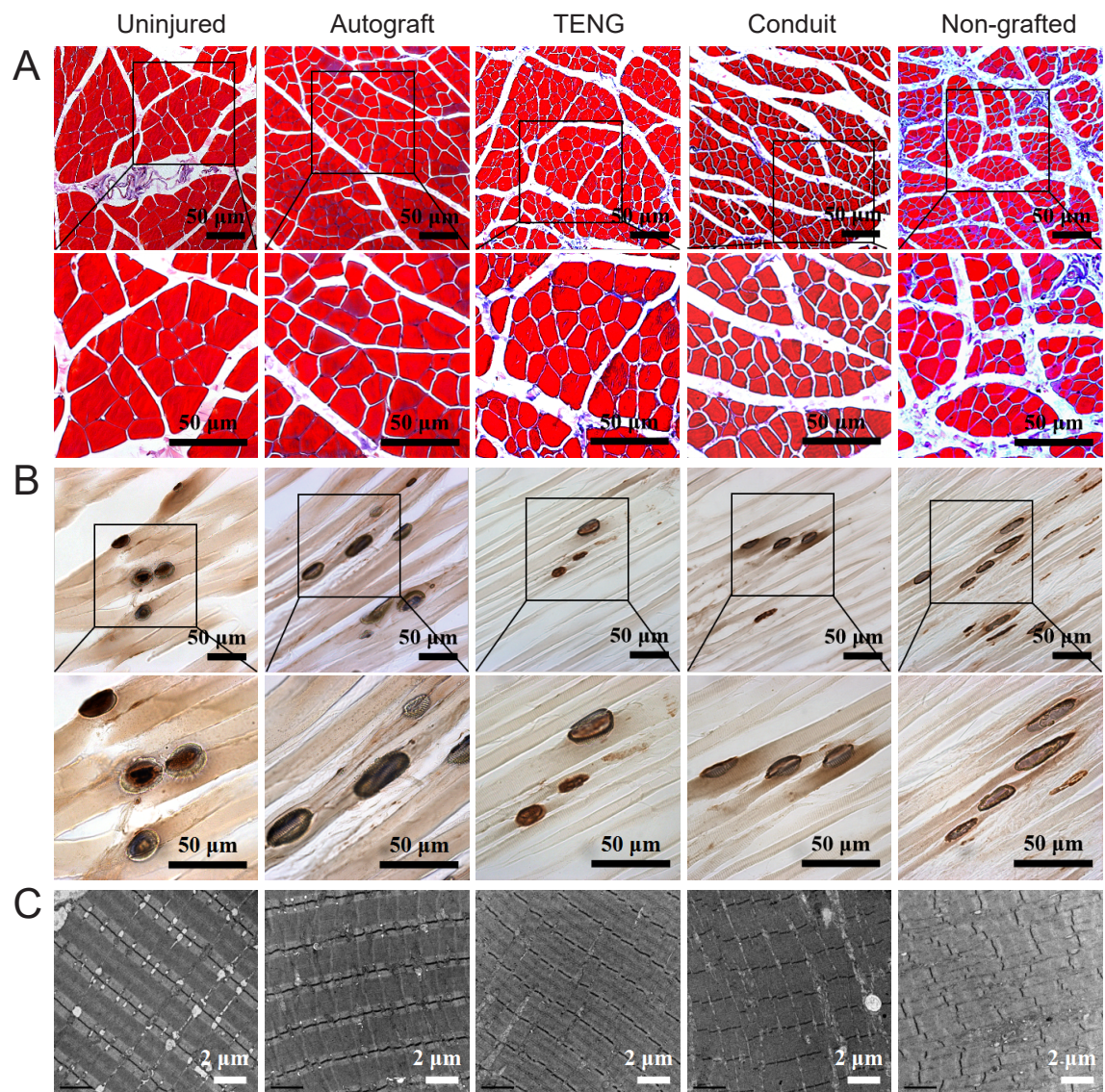

Supplement: Supplementary file 2 — Additional file 2: Figure S1. Histological observation of tibialis anterior muscle. a Representative images of masson trichrome staining of cross-sectional tibialis anterior muscle in uninjured, autograft, TENG, conduit and non-grafted groups. Scale bar, 50 μm. b Representative images of cholinesterase histochemistry staining for motor endplates of longitudinal tibialis anterior muscle section in uninjured, autograft, TENG, conduit and non-grafted groups. Scale bar, 50 μm. c Representative TEM images of muscle segment morphology of tibialis anterior muscle in uninjured, autograft, TENG, conduit and non-grafted groups. Scale bar, 2 μm. [file 13287_2019_1517_MOESM2_ESM.pdf]
